# Gait disorders in CKD patients: muscle wasting or cognitive impairment? A cross-sectional pilot study to investigate gait signatures in Stage 1–5 CKD patients

**DOI:** 10.1186/s12882-022-02697-8

**Published:** 2022-02-21

**Authors:** Damiano D. Zemp, Olivier Giannini, Pierluigi Quadri, Marco Rabuffetti, Mauro Tettamanti, Eling D. de Bruin

**Affiliations:** 1 Institute of Human Movement Sciences and Sport, Department of Health Sciences and Technology, ETH Zurich, Zurich, Switzerland; 2 Geriatric Service, Ospedale Regionale di Mendrisio, EOC, Mendrisio, Switzerland; 3 Department of Medicine, Ente Ospedaliero Cantonale, Bellinzona, Switzerland; 4 Division of Nephrology, Ente Ospedaliero Cantonale, Lugano, Switzerland; 5 Faculty of Biomedical Sciences, Università Della Svizzera Italiana, Lugano, Switzerland; 6 IRCCS Fondazione Don Carlo Gnocchi, Milano, Italy; 7 Department of Neuroscience, Istituto di Ricerche Farmacologiche Mario Negri IRCCS, Milano, Italy; 8 Department of Neurobiology, Care Sciences and Society, Karolinska Institute, Stockholm, Sweden; 9 OST – Eastern Swiss University of Applied Sciences, Department of Health, St. Gallen, Switzerland

**Keywords:** Functional decline, Chronic kidney disease, Gait disorders, Gait variability, Cognitive impairment, Sarcopenia

## Abstract

**Background:**

Instrumental gait analysis in nephrology is widely neglected, although patients with chronic kidney disease (CKD) show brain changes due to cerebrovascular disease and metabolic disorders that can potentially influence gait quality. Our study assesses the association between CKD stages and gait parameters, to understand the prevalent status of brain related gait parameters (i.e. variability) and of performance related parameters (i.e. gait speed, stride length). We hypothesize that gait changes are detectable already in early stages of CKD.

**Methods:**

Forty-five participants distributed in 5 CKD severity groups underwent an instrumental gait analysis via a triaxial accelerometer affixed to the lower trunk under single- and dual-task conditions. In addition to spatio-temporal parameters, variability and dual-task cost of gait were extracted. A battery of clinical assessments was conducted with the aim of helping to better explain the findings of the gait analysis. A correlation analysis was made to investigate a linear relation between gait parameters and CKD severity.

**Results:**

Statistically significant correlations (Pearson correlation coefficient) with CKD severity were found for gait speed (*p* < 0.01, *r* = -0.55, 95% CI [-0.73;-0.30]), stride length ( *p* < 0.01, *r* = -0.40, 95% CI [-0.62;-0.12]), step length (*p* < 0.01, *r* = -0.41, 95% CI [-0.63;-0.13], coefficient of variance (CV) of step length (*p* = 0.01, *r* = 0.36, 95% CI [0.08;0.59]), gait regularity (*p* < 0.01, *r* = -0.38, 95% CI [-0.61;-0.10]), dual-task cost of gait speed (*p* < 0.01, *r* = 0.40, 95% CI [0.13;0.62]) and dual-task cost of stride time (*p* = 0.03, *r* = 0.32, 95% CI [0.03;0.57]). Adjustment for age and gender confirmed all results except for gait regularity. With increasing severity of renal failure, Handgrip strength, Time for the Expanded Timed Get Up and Go test, executive functions, haemoglobin, and haematocrit, worsen.

**Conclusions:**

The correlation of CKD severity with spatio-temporal parameters (performance indices mainly relatable to peripheral functionality) and with variability of gait (related to central factors) supported by the results of the clinical assessments, suggests that gait disturbance in CKD patients is not only due to metabolic factors that lead to muscle wasting, but also to brain changes that affect motor control. This suggests that the treatment of renal disease should include cognitive aspects in addition to metabolic and functional factors.

**Supplementary Information:**

The online version contains supplementary material available at 10.1186/s12882-022-02697-8.

## Background

Patients at end stage renal disease (ESRD) are known to be particularly frail [1]. Compared to the general population, they show a reduced physical performance [2, 3], a higher fall rate [4–8], and higher cognitive impairment [9].

Often the lower health status of ESRD has been explained with factors related to the renal replacement therapy (RRT) – mostly haemodialysis (HD) – in particular, a reduced physical activity level [10–12], and cerebrovascular disorders [13, 14]. Although this explanation sounds reasonable, recently the transitional phase from a severe kidney failure to chronic haemodialysis was analysed in order to better understand the impact RRT has on the frailty process of CKD patients [15–17]. These reports share the observation that a frail population in the pre-dialysis phase or in the first months of HD seems to exhibit no further deterioration once the patients started their RRT.

Currently there is not much detailed knowledge about the highly prevalent gait impairments seen in CKD and ESRD patients, and how these impairments affect gait quality [18]. When gait assessment is applied to CKD patient populations it is rather limited to the dialytic population, and pre-dialytic groups are rather neglected in this regard, notwithstanding the suggestions that gait abnormalities that lead to heightened risk of falls already exist in early stages of CKD [4–8]. CKD patients show a significantly slower gait speed that is associated with physical [19–22], cognitive [23], sensory [24] and metabolic [25, 26] capacities, factors that are all influenced by CKD severity. However, cognitive factors may also impact gait in people with CKD [26, 27], and may be mediated by small vessel disease often seen in these patients [18]. A recent study found an association between gait abnormality and CKD severity [28], which suggests that gait is already affected in early stages of CKD and not only at stage 5. It is necessary to clarify whether changes in spatio-temporal gait variables become apparent, and whether these in turn relate to changes in cognition and fall events.

A cross-sectional study design will allow assessment of the relationship between CKD exposure and gait outcomes, and consequently help determine whether a longitudinal study would be warranted [29]. The aim of this study, therefore, is to analyse gait parameters in patients categorised into different CKD stages and specifically the presence of a worsening in these parameters as CKD progress. Because of the degenerative nature of CKD that includes the loss of muscle mass and the development of cognitive disorders with already mild reduction of the renal function, we hypothesise to find a linear decrease of gait quality in dependence of the CKD severity.

## Methods

### Study design

This is a cross-sectional observational study in an ambulant setting of 5 groups of CKD patients at different severity stages, based on the estimated glomerular filtration rate (eGFR) in accordance with K/DOQI guidelines [30] calculated with the CKD-EPI^1^ creatinine Eq. [31]. The study protocol included an instrumental gait analysis in a laboratory setting via a triaxial accelerometer affixed to the lower trunk, and a number of clinical assessments. Each patient underwent a single visit of about 90 min.

### Participants and setting

We recruited the patients in Canton Ticino – Switzerland, between July 2020 and May 2021. Medical doctors of two nephrology units of the multicentric public hospital (Ente Ospedaliero Cantonale, in the towns of Mendrisio and Lugano), asked patients with a kidney disease about their interest in participating in the study. In case of a positive answer, their contact data were sent to the principal investigator who called the patient and fixed the date for a visit. Before starting their visit, inclusion and exclusion criteria were checked, the patients were informed orally, received written information, and signed the written informed consent form. Patients with normal kidney function (stage 1) or mild to severe kidney dysfunction (stages 2–5), who were able to understand information for executing assessments, and could walk for about 20 m alone and without a walking aid, were eligible for the study. Patients with an unstable or preterminal health status (e.g. ongoing oncological treatment, recent surgery), diagnosis of depressive syndromes or dementia (Clinical Dementia Rate Scale ≥ 1 [32]) were excluded. Patients at stages 1 and 2 were inserted in the same group, as in neither stage the kidney disease influence relevantly organs homeostasis and represent a major health problem [30]. The influence of the haemodialytic therapy on the frailty process of CKD patients is not yet understood. This led us to analyse CKD 5 patients undergoing HD separately from CKD 5 patients not undergoing RRT.

### Variables

Gait was assessed following international guidelines that suggest to let the patient walk in a single- and a dual-task condition [33–35]. The instrumental gait analysis was performed in the hospital on a 14 m pathway via a triaxial accelerometer, designed for clinical gait analysis in a laboratory setting and validated for patients with chronic conditions [36, 37] (DynaPort MiniMod, McRoberts, The Hague, NL), affixed by an elastic belt to the lower trunk, between the left and the right spina iliaca posterior superior [38]. The inverted pendulum model was used to calculate spatio-temporal gait parameters [39, 40]. Participants walked two times under single-task (ST) and two times under dual-task (DT) conditions (counting backward from 100 in steps of 3) over the pathway. The calculation of stride variability and gait regularity requires the patients to walk at a steady state speed; therefore, the first and the last 2 m of the reading were excluded from the analysis, meaning that between 15 and 35 strides were used for calculating gait parameters, depending on the step length of each participant.

In addition to the spatio-temporal parameters (stride and step time, stride and step length, gait speed and cadence), variability was assessed using the coefficient of variation (CV = standard deviation / mean) of stride and step both in the spatial and the temporal domain, and gait regularity (autocorrelation analysis of the acceleration module, i.e. norm of the acceleration vector) [41]. Dual-task cost (in percent) was calculated for gait speed, cadence, and stride length (1-ST_value_/DT_value_, since this was expected to be lower in the dual-task condition) and for stride time (ST_value_/DT_value_—1, since this was expected to be lower in the single-task condition).

For the description of the population, several clinical tests were assessed. A detailed description can be found in Additional file 1.

### Statistical analysis

Since previous studies gave a hint of the presence of at least a moderate inverse correlation (Pearson's rho > 0.50) between increasing CKD severity and worsening in motoric [18, 42] and cognitive functions [43], we aimed at reaching a target of 50 patients (almost equally distributed across the five severity groups) to estimate a 95% confidence interval (CI) of a Pearson's r linear correlation coefficient, with a lower limit 0.24, and an upper limit 0.18, from the estimated correlation index (i.e. a 95% CI ranging from 0.26 to 0.68 in case of a point estimate of *r* = 0.50).

Demographic, clinical, functional and gait characteristics of the subjects were described using mean and standard deviation, and reporting minimum and maximum values, for numerical variables, while sex was categorised numerically.

Although the CKD stage is not strictly a continuous variable, the number of stages (five) of CKD severity is relatively high, and previous projects were carried out using this approximation [44]. Due to the degenerative nature of CKD (i.e. subjects transition from a mild status to more serious conditions and not vice versa), we tentatively considered the relationship between kidney function and gait speed to be approximately linear or at least monotone, so we analysed the data using a Pearson's linear correlation coefficient r, reporting its estimate and 95% CI. We also performed the same analyses by estimating Spearman's r_s_ coefficient to confirm the existence of associations by relaxing the prerequisites of the parametric Pearson's r coefficient. Spearman's r_s_ 95% CIs were obtained using a bias corrected bootstrap method with 1000 replications. The association between CKD and gait variables was studied, also controlling for age and sex, performing separate linear regressions for each gait variable: in this case a corrected regression coefficient (increase/decrease in gait variable value for a CKD class worsening) was reported. We did no include other possibly important covariates in the adjusted model due to the relatively low number of the collective, since including more variable could create multicollinearity problems.

All participants completed the gait analysis and for all 45 participants all data are available. Missing data for clinical aspects (i.e. for technical issues) were not replaced and no adjustment of mean and standard deviation was carried out. We checked for the presence of outliers calculating a Mahalanobis distance on gait variables: one subject had a distance which was almost double relative to the second one. After inspecting the groups of variables, we found that the distance was almost entirely due to his dual task performance, where his Mahalanobis distance was almost five times the second one, therefore we decided to exclude him from parametric analyses in dual task analyses only, while retaining him in non-parametric analyses (where, rather obviously, no difference was found with or without his presence).

Significance cut-off was set at *p* ≤ 0.05. Statistical analyses were performed using Stata 15.1 (StataCorp) and JMP Pro 15 (SAS Institute Inc.).

## Results

 51 patients were asked by the nephrologist to participate. 1 person (CKD 5 not on HD) died before the principal investigator could fix the date for the visit. 5 patients change their mind once contacted by the principal investigator and denied to participate (1 was CKD 3, 2 were CKD 4 and 2 were CKD 5 not on dialysis). No participant was excluded by the principal investigator during the visit. 45 patients accepted to participate in the study which led to an almost equal distribution of the participants in 5 CKD severity groups: stage 1–2 (*n* = 8); stage 3 (*n* = 9); stage 4 (*n* = 9); stage 5 not on dialysis (*n* = 10); 5 on HD (*n* = 9). The combined CKD group 1 & 2 was on average younger than the other three groups (60 vs. 74 years). Table 1 describes the clinical aspects of the participants, and shows a general tendency to worse clinical status, in particular a reduction of physical performance, with decreasing eGFR. Table 2 reports descriptive values for single gait characteristics by CKD status. Table 3 reports the correlation and regression analyses on the single characteristics. A decrease in gait speed, stride length and step length, among the spatio-temporal parameters, was statistically associated with a worsening kidney function, both at a univariable and at a multivariable analysis. Among parameters evaluating variability only CV of step length was correlated in univariable and age and sex corrected models. CV of stride time was significantly correlated only in non-parametric analysis and gait regularity was correlated only at univariable analyses.

**Table 1:** Main demographic, clinical and functional data: mean ± SD [range]

	**CKD 1–2** **(*n* = 8)**	**CKD 3** **(*n* = 9)**	**CKD 4** **(*n* = 9)**	**CKD 5** **(*n* = 10)**	**CKD 5 (HD)** **(*n* = 9)**	**Reference** **value**
**General characteristics**						
Gender (M / W)	6 / 2	6 / 3	8 / 1	4 / 6	4 / 5	
Age (years)	60.4 ± 13.7 [40–83]	74.7 ± 9.1 [63–92]	77.1 ± 10.5 [53–85]	72.1 ± 4.4 [65–79]	75.9 ± 7.2 [62–86]	
BMI (kg/m^2^)	25.3 ± 2.4 [22–28.7]	27.4 ± 4.0 [22.2–33.7]	28.4 ± 3.7 [23.5–36.4]	28.9 ± 5.5 [16.7–34.3]	29.2 ± 3.3 [22.6–35.1]	18.5 – 24.9
Schooling (years)	11.5 ± 3.0 [8–16]	9.6 ± 2.6 [5–14]	10.1 ± 4.0 [5–17]	6.9 ± 2.8 [3–13]	8.7 ± 3.3 [5–17]	
**CKD specific parameters**						
eGFR (ml/min/1.73 m^2^)	68.8 ± 16.2 [56–108]	35.1 ± 5.9 [26–44]	22.1 ± 4.9 [16–29]	10.3 ± 1.7 [7–13]	9.4 ± 4.0 [4–17]	
Time on HD (weeks)	-	-	-	-	81 ± 43 [13–131]	
**Health status**						
Physical health	52.1 ± 7.7 [36.5–60.9]	45.5 ± 10.1 [25.0–55.3]	49.7 ± 8.6 [34.2–62.4]	40.3 ± 7.8 [25.0–52.5]	37.6 ± 10.2 [19.9–50.6]	> 40
Mental health	53.8 ± 7.0 [40.0–60.7]	52.7 ± 7.9 [38.1–62.0]	49.5 ± 12.9 [23.3–60.6]	54.4 ± 7.9 [37.9–65.1]	48.7 ± 8.0 [39.3–59.7]	> 40
Autonomy	100 ± 0.0 [100–100]	99.4 ± 1.7 [95–100]	100 ± 0.0 [100–100]	98.5 ± 3.4 [90–100]	91.7 ± 8.3 [80–100]	≥ 75
Independence	21.0 ± 1.5 [18–22]	20.4 ± 1.4 [17–22]	18.4 ± 1.6 [16–21]	17.1 ± 3.2 [10–22]	12.8 ± 3.9 [7–19]	≥ 17
General fatigue	6.0 ± 1.9 [4–9]	10.4 ± 4.2 [4–16]	9.6 ± 3.8 [5–17]	11.5 ± 3.1 [7–15]	11.1 ± 4.4 [4–17]	< 10
Pain	17.5 ± 27.1 [0–80]	17.8 ± 18.4 [0–50]	13.9 ± 18.2 [0–50]	51.0 ± 33.8 [0–100]	29.4 ± 24.8 [0–60]	< 40
GDS-10	0.9 ± 0.6 [0–2]	1.1 ± 1.2 [0–4]	2.6 ± 2.9 [0–7]	2.6 ± 3.4 [0–10]	3.7 ± 1.7 [1–6]	≤ 5
Comorbidity severity Index	2.0 ± 0.1 [1.8–2.0]	2.5 ± 0.3 [2.3–3.0]	2.2 ± 0.3 [1.7–2.5]	2.0 ± 0.2 [1.6–2.2]	2.0 ± 0.3 [1.5–2.5]	≤ 2
Comorbidity index	0.0 ± 0.0 [0–0]	1.3 ± 0.5 [1, 2]	1.7 ± 1.1 [1–4]	1.6 ± 1.1 [1–4]	1.6 ± 0.5 [1, 2]	≤ 2
**Physical Performance**						
SPPB	11.1 ± 1.1 [9–12]	11.6 ± 0.7 [10–12]	10.3 ± 1.3 [8–12]	9.4 ± 2.5 [4–12]	6.3 ± 2.4 [3–10]	> 6
ETGUG (seconds)	18.7 ± 1.8 [16.3–21.4]	20.7 ± 3.7 [14.8–26.7]	22.6 ± 3.3 [16.9–26.3]	27.5 ± 9.6 [16.4–43.8]	36.7 ± 13.5 [20.5–55.6]	< 34
POMA	27.0 ± 0.9 [26–28]	26.0 ± 2.3 [21–28]	25.6 ± 1.9 [23–28]	24.7 ± 2.8 [21–28]	21.7 ± 4.4 [16–28]	> 19
Steps/day^a^	10,876 ± 3,419 [5,843–16,881]^1^	9,979 ± 5,432 [3,664–22,027]	6,251 ± 2,779 [3,576–11,463]^1^	7,144 ± 3,639 [2,839–15,862]	3,406 ± 3,257 [402–11,145]	> 5,000
Handgrip (kg)^b^ -Male -Female	47.7 ± 9.0 [38–58] 36.0 ± 4.2 [33–39]	41.7 ± 13.1 [21–58] 28.0 ± 0.0 [28–28]	29.0 ± 7.6 [20–44] 24.0 ± 0.0 [24–24]	28.3 ± 9.6 [19–40] 10.4 ± 3.3 [8–16]	21.3 ± 4.3 [15–25] 12.4 ± 3.9 [8–18]	≥ 27 ≥ 16
Hip flexion (kg)^c^ -Male -Female	23.0 ± 6.9 [14.0–31.3] 16.1 ± 0.9 [15.4–16.7]	23.2 ± 7.6 [18.8–36.7] 11.9 ± 1.5 [10.1–12.9]	19.6 ± 3.1 [14.6–23.2] 12.0 ± 0.0 [12.0–12.0]	18.9 ± 13.5 [9.3–42.4] 14.3 ± 2.1 [11.4–17.4]	12 ± 3.6 [8.2–15.3] 11.4 ± 3.4 [7–15.3]	> 11 > 10
**Cognitive Status**						
MMSE	28.9 ± 1.1 [27–30]	28.3 ± 0.7 [27–29]	26.7 ± 2.3 [23–29]	26.2 ± 4.1 [16–29]	25.9 ± 1.6 [24–28]	> 24
FAB^d^	3.3 ± 0.7 [2–4]	2.9 ± 1.1 [1–4]	0.7 ± 1.1 [0–3]	1.9 ± 1.5 [0–4]	1.3 ± 1.5 [0–4]	≥ 1
TMT_A^d^	3.8 ± 0.7 [2–4]	3.3 ± 1.1 [1–4]	2.2 ± 1.8 [0–4]	2.2 ± 1.9 [0–4]	2.7 ± 1.6 [0–4]^1^	≥ 1
TMT_B^d^	3.6 ± 0.7 [2–4]	2.7 ± 1.7 [0–4]	2.8 ± 1.9 [0–4]	2.3 ± 2.1 [0–4]^1^	1.7 ± 2.0 [0–4]^1^	≥ 1
**Haematology parameters**						
Calcium (mmol/L)	2.4 ± 0.1 [2.3–2.5]^2^	2.3 ± 0.1 [2.2–2.4]^1^	2.3 ± 0.2 [2.0–2.5]^1^	2.3 ± 0.2 [2.0–2.5]	2.2 ± 0.1 [2.0–2.4]	2.15 – 2.55
Phosphates (mmol/L)	1.0 ± 0.2 [0.8–1.2]^2^	1.1 ± 0.1 [0.9–1.3]^1^	1.2 ± 0.2 [0.9–1.4]^1^	1.6 ± 0.2 [1.3–1.8]	1.7 ± 0.5 [1.0–2.9]	0.81 – 1.45
Haemoglobin (g/L)	142.3 ± 9.5 [124–154]^1^	133.8 ± 13.6 [115–156]	130.0 ± 20.1 [99–171]^1^	101.5 ± 10.5 [84–119]	103.8 ± 11.2 [87–122]	140 – 180
Haematocrit (L/L)	0.44 ± 0.03 [0.38–0.48]^1^	0.41 ± 0.05 [0.36–0.50]	0.40 ± 0.07 [0.29–0.53]^1^	0.31 ± 0.04 [0.26–0.38]	0.31 ± 0.04 [0.26–0.39]	0.45 – 0.55

*BMI* Body Mass Index, *eGFR* estimated glomerular filtration rate, *GDS* Geriatric Depression Scale, *SPPB* Short Physical Performance Battery, *ETGUG* Expanded Timed Get up and Go Test, *POMA* Performance Oriented Mobility Assessment, *MMSE* Mini-Mental State Examination, *FAB* Frontal Assessment Battery, *TMT* Trail Making Test. ^a^Amount of steps per/day were recorded with a pedometer, the participants had to wear for a week (Step Watch™, Modus, Washington DC, USA). ^b^Handgrip was tested using a Jamar® hydraulic hand dynamometer (Performance Health International LTD, Sutton-in-Ashfiled, UK). ^c^Hip flexion was tested using a manual muscle tester (Nicholas MMT, Model 01,160, Lafayette Instrument, Lafayette, USA). ^d^For FAB and TMT, the Equivalent Score was calculated adjusting the tests score for age and schooling.﻿ ^1^The value of 1 patient is missing. ^2^The values of 2 patients are missing

**Table 2:** Gait characteristics: mean ± SD [range]

	**CKD 1–2** **(*n* = 8)**	**CKD 3** **(*n* = 9)**	**CKD 4** **(*n* = 9)**	**CKD 5** **(*n* = 10)**	**CKD 5 (HD)** **(*n* = 9)**
**Spatio-temporal parameters**					
Gait speed (m/s)	1.31 ± 0.14 [1.09–1.55]	1.22 ± 0.20 [0.86–1.53]	1.12 ± 0.15 [0.93–1.36]	1.03 ± 0.36 [0.56–1.54]	0.86 ± 0.25 [0.53–1.23]
Cadence (steps/min)	107.4 ± 9.0 [95–120]	113.7 ± 10.5 [103–136]	110.0 ± 6.0 [98–118]	110.8 ± 14.6 [84–130]	101.6 ± 13.7 [79–126]
Stride time (s)	1.14 ± 0.09 [1.03–1.27]	1.08 ± 0.08 [0.95–1.18]	1.10 ± 0.06 [1.02–1.22]	1.12 ± 0.16 [0.95–1.46]	1.21 ± 0.17 [0.96–1.55]
Stride length (m)	1.37 ± 0.26 [1.11–1.78]	1.18 ± 0.16 [0.96–1.42]	1.18 ± 0.25 [0.67–1.56]	1.08 ± 0.32 [0.74–1.59]	1.05 ± 0.22 [0.81–1.44]
Step time (s)	0.57 ± 0.04 [0.52–0.63]	0.54 ± 0.04 [0.48–0.59]	0.55 ± 0.03 [0.51–0.61]	0.56 ± 0.08 [0.47–0.73]	0.61 ± 0.08 [0.48–0.77]
Step length (m)	0.69 ± 0.13 [0.56–0.89]	0.59 ± 0.08 [0.48–0.71]	0.59 ± 0.13 [0.33–0.78]	0.54 ± 0.16 [0.37–0.79]	0.52 ± 0.11 [0.4–0.72]
**Variability**					
CV Stride time	3.0 ± 1.5 [1.2–5.5]	4.1 ± 3.4 [1.3–10.7]	2.8 ± 1.6 [0.7–6.1]	4.9 ± 2.8 [1.4–10.7]	4.4 ± 1.5 [1.8–6.9]
CV Stride length	3.4 ± 1.0 [1.4–5.2]	5.4 ± 3.4 [2.6–13.1]	4.9 ± 2.8 [2.3–10.4]	7.5 ± 4.1 [3.0–15.4]	4.5 ± 2.0 [1.6–8.2]
CV Step time	9.0 ± 7.4 [2.3–18.7]	7.6 ± 4.8 [2.1–16.9]	7.4 ± 3.7 [2.2–12.7]	10.2 ± 4.3 [3.3–16.8]	7.9 ± 2.8 [2.6–12.0]
CV Step length	6.5 ± 3.1 [4.0–13.1]	7.3 ± 4.0 [4.5–16.2]	8.3 ± 3.4 [4.8–15.7]	11.1 ± 5.3 [3.9–19.9]	11.3 ± 8.0 [2.7–30.4]
Gait regularity	0.96 ± 0.03 [0.91–0.99]	0.95 ± 0.04 [0.87–1.00]	0.90 ± 0.16 [0.47–0.98]	0.92 ± 0.07 [0.78–0.99]	0.83 ± 0.13 [0.53–0.95]
**Dual-task cost of gait**					
Gait speed (%)	11.8 ± 14.2 [2–45]	11.6 ± 10.3 [-4–28]	14.6 ± 9.2 [2–29]	15.8 ± 10.2 [-1–35]	19.3 ± 10.7 [8–37]
Cadence (%)	7.9 ± 11.6 [-1–35]	6.8 ± 7.4 [-1–21]	6.4 ± 5.7 [-2–16]	7.8 ± 4.8 [-1–14]	10.0 ± 8.4 [-1–25]
Stride time (%)	11.8 ± 22.6 [-1–67]	7.4 ± 7.9 [-2–21]	8.0 ± 6.8 [-2–19]	8.9 ± 5.6 [-1–17]	12.3 ± 11.3 [0–34]
Stride length (%)	-1.8 ± 4.5 [-7–6]	2.4 ± 6.6 [-5–15]	3.1 ± 8.8 [-11–17]	-1.2 ± 9.9 [-14–18]	4.7 ± 8.2 [-6–23]

*CV* Coefficient of variation

**Table 3:** Correlation coefficient, p value and confidence interval between gait parameters and CKD severity (*n* = 45)

	**Pearson' r** **(*p* value) [95% CI]**	**Spearman's r**_**s**_ **(*p* value) [95% CI]**	**Regression coefficient** **(*p* value) [95% CI]**
**Spatio-temporal parameters**			
Gait speed (m/s)	**-0.55 (*p* < 0.01) [-0.73;-0.30]**	**-0.53 (*p* < 0.01) [-0.72;-0.28]**	**-0.07 (*p* < 0.01) [-0.12;-0.02]**
Cadence (steps/min)	-0.18 (*p* = 0.23) [-0.45;0.12]	-0.14 (*p* = 0.34) [-0.44;0.16]	-0.51 (*p* = 0.70) [-3.2;2.2]
Stride time (s)	0.22 (*p* = 0.15) [-0.08;0.48]	0.15 (*p* = 0.33) [-0.20;0.43]	0.01 (*p* = 0.52) [-0.02;0.04]
Stride length (m)	**-0.40 (*p* < 0.01) [-0.62;-0.12]**	**-0.39 (*p* < 0.01) [-0.62;-0.13]**	**-0.06 (*p* = 0.05) [-0.11;-0.001]**
Step time (s)	0.21 (*p* = 0.16) [-0.09;0.47]	0.14 (0.37) [-0.17;0.44]	0.004 (*p* = 0.55) [-0.01;0.02]
Step length (m)	**-0.41 (*p* < 0.01) [-0.63;-0.13]**	**-0.39 (*p* < 0.01) [-0.62;-0.10]**	**-0.03 (*p* = 0.04) [-0.06;-0.001]**
**Variability**			
CV Stride time	0.22 (*p* = 0.18) [-0.08;0.48]	**0.29 (*p* = 0.05) [0.02;0.52]**	0.29 (*p* = 0.31) [-0.28;0.86]
CV Stride length	0.19 (*p* = 0.20) [-0.11;0.46]	0.22 (*p* = 0.14) [-0.05;0.45]	0.34 (*p* = 0.37) [-0.41;1.10]
CV Step time	0.02 (*p* = 0.90) [-0.28;0.31]	0.13 (*p* = 0.39) [-0.22;0.42]	0.36 (*p* = 0.52) [-0.76;1.48]
CV Step length	**0.36 (*p* = 0.01) [0.08;0.59]**	**0.37 (*p* = 0.01) [0.06;0.64]**	**1.42 (*p* = 0.02) [0.21;2.62]**
Gait regularity	**-0.38 (*p* < 0.01) [-0.61;-0.10]**	**-0.48 (*p* < 0.01) [-0.69;-0.23]**	-0.02 (*p* = 0.06) [-0.05;0.001]
**Dual-task cost of gait**^**a**^			
Gait speed (%)	**0.40 (*p* < 0.01) [0.13;0.62]**	**0.34 (*p* = 0.02) [0.03;0.59]**	**2.52 (*p* = 0.03) [0.32;4.72]**
Cadence (%)	0.28 (*p* = 0.07) [-0.02;0.53]	0.20 (*p* = 0.19) [-0.09;0.50]	1.10 (*p* = 0.13) [-0.35;2.55]
Stride time (%)	**0.32 (*p* = 0.03) [0.03;0.57]**	0.26 (*p* = 0.09) [-0.04;0.54]	1.71 (*p* = 0.06) [-0.05;3.47]
Stride length (%)	0.12 (*p* = 0.43) [-0.18;0.40]	0.15 (*p* = 0.34) [-0.13;0.40]	0.85 (*p* = 0.36) [-0.99;2.70]

^a^*n* = 44 for Pearson's r and linear regression

Among parameters on dual-task cost of gait, gait speed was correlated in all three models, while stride time was correlated only in parametric univariable analysis. In Additional file 2, the data are presented graphically with Boxplots.

## Discussion

The aim of this study was to analyse gait parameters in patients categorised into different CKD disease stages and to determine whether these measures got worse in relation to the disease progression. To the best of our knowledge, this is the first study that focused on the correlation between several gait variability parameters and CKD severity. The results indicate that three variability parameters out of five (CV of stride time, CV of step length and gait regularity) deteriorate in a linear way with the reduction of eGRF and suggests that already in early stages of CKD (namely stage 3) changes in the brain structure may influence gait quality. The other variability measures show no decline over the disease progression, however, the values observed for step time and stride length variability show values that are consistently high and are, when compared to benchmarks, to be considered in ranges attributable to pathologic walking behaviour [45]. It can, therefore, be speculated that ceiling effects may explain why further worsening in these parameters cannot be detected.

It is widely known that CKD severity affects the health status both for physical and cognitive performance [1–3, 9] and the clinical parameters collected in this study confirms this. The relation between muscle strength and gait performance is well known [20, 26, 46] and decreased muscle strength may explain why people show decreased walking velocity [47]. Therefore, it is not surprising to find in our results the same tendency towards a worsening result with increasing CKD severity for clinical muscle related parameters (handgrip strength, fatigue, physical health, time for ETGUG) as well as for gait speed and stride length. Less studied in this population, however, is the relation between cognitive aspects and gait variability parameters.

Gait disorders may be caused by muscular peripheral impairment, may have a neurologic central origin, or may be due to a combination of factors [48]. Where lower extremity strength would be indicative for how fast people can walk [49], measures of gait variability are indicative of brain functioning [50]. The proxy assessments related to skeletal muscle mass that we applied (ETGUG, handgrip, fatigue) showed a deterioration already in early stages of the illness, and confirm that muscle wastage is a driving factor for decreased walking speed in CKD, and should, therefore, be monitored throughout all stages [51, 52]. In fact, it is known that CKD-sarcopenia is a secondary sarcopenia, which, compared to the age-related primary sarcopenia, occurs earlier and in a more intense way and with a greater magnitude [52]. This seems to be confirmed in our sample, where the difference in muscle strength related factors (i.e. handgrip) between age-matched patients at different CKD stages is meaningful. This rapid loss of muscle strength is explained by mitochondrial damage and protein degradation [52–54] typical for CKD patients. However, changes in grip strength are largely reflective of decreased integrity of the nervous system [55] and sarcopenia is also linked to changes in the central nervous system [56, 57]. This would hint to the possibility of gait disorders in CKD caused by peripheral and central factors.

Although these results apparently also explain the slowdown of gait speed and shortening of stride length [58, 59], some other factors, e.g. cognitive factors, should be considered as well. These factors may help in explaining the relation between gait performance, brain health and kidney failure [60]. In particular, cerebrovascular diseases are related to a reduced gait performance [61–64]. These findings led to a definition of the motoric cognitive risk syndrome [65, 66] as characterized by a slow gait and mild cognitive impairment. The CKD population is more affected by cerebrovascular disease, and the rate of vascular dementia is higher than the rate of degenerative dementia, in contrast to the general population [67], which should be reflected in gait variability values. The individuals selected for our study show in general values for gait variability in all five disease stages that are indicative of pathological walking [45]. The reduction of both gait performance (lower gait speed and shorter stride length) and executive function (FAB, TMT) in CKD stages ≥ 3 observed in our study is therefore coherent with brain changes due to kidney failure [68–70]. The higher dual-task cost of gait speed is an additional hint towards this link [71–74]. This finding confirms the association between gait disorders and gray matter atrophy in CKD patients both with and without cognitive disorders [75]. Based on these observations a more comprehensive analysis of gait changes in clinical CKD populations seems warranted.

In our study we also calculated gait variability, that is known to be related to neurocognitive factors [76–80]. As gait variability and gait speed are controlled by different brain regions [78, 79], and gait speed is also affected by muscle strength (see above), it is not unexpected that they are influenced differently by CKD severity, and that they are not related [81, 82]. Finally, in addition to metabolic disorders that lead to muscle mass loss and changes in the brain that lead to gait impairment, it is worth mentioning that recent research showed a disturbed corticospinal control of gait in sarcopenic patients [83]. Functional decline of gait in CKD patients seems a complex process that should be treated through a multidisciplinary approach.

The aim of this study was to describe gait characteristics of CKD patients at different stages. We found statistically relevant correlations between a decline in gait speed, stride length and spatial and temporal variability. This can be explained on the one hand by muscle wastage, and on the other hand by cognitive decline, especially executive function, due to cerebrovascular disorders. As these changes start already in patients with a moderately reduced kidney function, interventions to prevent cognitive and physical decline should be offered early in form of training that stimulates both physical and cognitive domains.

## Strength and limitations

Our study is one of the first describing quantitatively the gait characteristics of CKD patients through all disease stages and should, therefore, be considered as a pilot study and our conclusion treated as exploratory. Firstly, the small number of participants in each group has the risk that a few outliers (see Additional file 2) could have influenced the statistical significance. The small collective also prevented us from analysing the data taking into consideration other important modifiers and confounders. Secondly, the less restrictive selection criteria lead to a heterogenous group with participants with very different diagnoses. The third limitation relates to the cross-sectional study design. Where this design was helpful to reveal information about the prevalence of gait measures in CKD we should be prudent in deriving causal inferences [84]. Despite these limitations we found significant correlations between gait characteristics and CKD severity consistent with the clinical data collected, and in line with the literature that induce us to consider cognitive aspects, in addition to metabolic and physical performance aspects, in the treatment of patients with kidney failure long before the illness reaches a severe stage.

## Conclusions

Both a reduction in gait performance (gait speed and stride length) and an increase in gait variability and dual-task cost of walking were found to be correlated with CKD severity. This finding confirms on the one hand the influence of metabolic changes in the muscle that leads to muscle wastage and, in turn, to a reduced physical walking speed performance, together with a higher degree of fatigue recorded in the participants with CKD ≥ 3. On the other hand, the poor results shown in neurocognitive tests, in dual-task cost of gait speed analysis, and in variability measures of gait in participants with a more impaired renal function, hint towards brain changes that influence gait quality of CKD patients. These results suggest that the treatment of CKD patients should be multidisciplinary and should take cognitive aspects into consideration in addition to the metabolic and muscle strength functional properties even though they were obtained in a relatively small sample of subjects and therefore deserve to be confirmed by other (possibly larger) studies. Longitudinal studies exploring the development of gait in CKD patients over time are warranted.

## Supplementary Information

**Additional file 1. **

## Data Availability

The datasets used and/or analysed during the current study are available from the corresponding author on reasonable request.

## Abbreviations

BMI: Body Mass Index
CI: Confidence Interval
CKD: Chronic Kidney Disease
CV: Coefficient of variation
DT: Dual-task
eGFR: Estimated Glomerular Filtration Rate
ESRD: End Stage Renal Disease
ETGUG: Expanded Timed Get Up and Go Test
FAB: Frontal Assessment Battery
GDS-10: Geriatric Depression Scale
HD: Haemodialysis
K/DOQI: Kidney Disease Outcomes Quality Initiative
M: Men
MMSE: Mini-Mental State Examination
POMA: Performance Oriented Mobility Assessment
r: Pearson’s correlation coefficient
RRT: Renal Replacement Therapy
r_s_: Spearman’s correlation coefficient
SD: Standard Deviation
SPPB: Short Physical Performance Battery
ST: Single-task
TMT: Trail Making Test
W: Women

## Footnotes

1 Authors’ note: Chronic Kidney Disease Epidemiology Collaboration.
