# Supplementary material for: Gait disorders in CKD patients: muscle wasting or cognitive impairment? A cross-sectional pilot study to investigate gait signatures in Stage 1–5 CKD patients
Source: BMC Nephrol. 2022 Feb 21;23:72. doi: 10.1186/s12882-022-02697-8 (PMC8862207; doi:10.1186/s12882-022-02697-8)
Supplement: Supplementary file 1 — Additional file 1. [file 12882_2022_2697_MOESM1_ESM.docx]

Additional files

**Additional file 1:** Clinical assessments

| **Assessment** | **Abbreviation** | **Unit** | **Reference value** |
| --- | --- | --- | --- |
| Expanded Timed Get-up-and-Go Test | ETGUG | s | < 34 |
| Performance Oriented Mobility Assessment | POMA | 0 – 28 points | > 19 |
| Short Physical Performance Battery | SPPB | 0 – 12 points | > 6 |
| Handgrip | HG | kg | ≥ 27 (male)  ≥ 16 (female) |
| Hip Flexion | HF | kg | > 11 (male)  > 10 (female) |
| Weekly monitoring of physical activity | - | steps/day | > 5000 |
| The Short Form health survey | SF-12 | 24 – 57  19 – 61 points | > 40 (physical)  > 40 (mental) |
| The Barthel Index of activities of daily living | ADL | 0 – 100 points | ≥ 75 |
| Nottingham Extended Activities of Daily Living Scale | EADS | 0 – 22 points | ≥ 17 |
| Visual Analog Scale for pain | VAS-P | 0 – 100 points | < 40 |
| Geriatric Depression Scale | GDS-10 | 0 – 10 points | < 4 |
| Multidimensional fatigue inventory | MFI-20 | 0 – 24 points | > 10 |
| Mini-Mental State Examination | MMSE | 0 – 30 points | > 24 |
| Frontal Assessment Battery | FAB | 0 – 4 points* | ≥ 1 |
| Trail Making Test | TMT | 0 – 4 points* | ≥ 1 |
| Cumulative illness rating scale | CIRS-S  CIRS-C | 0 – 4  0 – 14 points | ≤ 2 (severity)  ≤ 2 (comorbidity) |
| Haematology parameters   - Calcium - Phosphates - Haemoglobin - Haematocrit | Ca^++^  PO_4_^-^ | mmol/L  mmol/L  g/L  L/L | 2.15 – 2.55  0.81 – 1.45  140 – 180  0.45 – 0.55 |
| * For FAB and TMT, the Equivalent Score was calculated adjusting the test score for age and schooling (1). | | | |

**Expanded Timed Get-up-and-Go Test (ETGUG) (2)**

The test measures the time required to stand up, walk 10 m, turn, walk back, and sit down.

**Performance Oriented Mobility Assessment (POMA) (3, 4)**

POMA is a task- oriented test that measures an older adult's gait and balance abilities. Scoring of the Tinetti Assessment Tool is done on a three-point ordinal scale with a range of 0 to 2. A score of 0 represents the most impairment, while a score of 2 represents independence. The individual scores are then combined to form three measures; overall gait assessment score, overall balance assessment score, and combined gait and balance score. The maximum score for the gait component is 12 points. The maximum score for the balance component is 16 points. The maximum total score is 28 points.

**Short Physical Performance Battery (SPPB) (5, 6)**

The SPPB includes tests of walking speed, standing balance and chair stand. Each SPPB component test (gait, balance and chair stand) is scored from 0 to 4, with a score of 0 representing inability to carry out the test, and 4 as the best performance. For balance, the participants are asked to maintain their feet side-by-side, in semi-tandem and tandem positions for 10 s each. For gait, a 4m walk at the participants’ usual speed was timed with a standing start. For the chair stand test, participants were asked to stand up and sit down five times as quickly as possible.

**Handgrip (HG)** (7, 8)

Handgrip strength was tested using a Jamar^®^ hydraulic hand dynamometer (Performance Health International LTD, Suton-in-Ashfield, UK).

**Hip flexion (HF)** (9, 10)

Hip flexion strength was tested using a Nicholas manual muscle tester (Model 01160, Lafayette Instrument, Lafayette, USA).

**Weekly monitoring of physical activity** (11-14)

The patients received a pedometer (Step Watch^TM^, Modus, Washington DC, USA) for 7 consecutive days that objectively measured their physical activity level. The device was attached to the right ankle and measured the number of right steps during the defined period. The number of right steps was doubled in order to have an output comparable with international normative data.

**Short Form health survey (SF-12) (15-17)**

The SF-12 is a commonly used instrument to measure the health-related quality of life at various ages. The instrument uses 12 questions to measure functional health and well-being from the patient’s perspective. The generic health-status measure reproduces the physical component summary (PCS) score and the mental component summary (MCS) score. The SF-12 is validated for long dialysis patients.

**Barthel Index of activities of daily living (ADL) (18, 19)**

The ADL assesses functional disability by quantifying patient performance in 10 activities of daily life. Scoring is done on a three-level scale: 0 (unable), 5 (needs help) and 10 (independent). The maximum score is 100.

**Nottingham Extended Activities of Daily Living Scale (EADS)** (20)

The EADS is used for assessing independence and includes 4 sections (mobility, kitchen, domestic and leisure) for a total of 22 items measuring the capacity to do a specific task. A score of 0 means the person needs help and 1 means the person is independent. The maximum score is 22.

**Visual Analog Scale for pain (VAS-P)** (21, 22)

The VAS-P is a widely used tool to measure subjective acute and chronic pain on a linear scale (100 mm line) that scores the pain between 0 (no pain) to 100 (worst pain).

**Geriatric Depression Scale (GDS-10)** (23, 24)

The GDS-10 is a screening test for depression in the elderly population, but validated also for younger adults. The scale is a 10-item, self-reporting instrument that uses Yes (1 points) and No (0 points) answers. A total score of 0 means no signs of depression.

**Multidimensional fatigue inventory (MFI-20)**(25, 26)

The MFI-20 is a 20-item scale designed to evaluate five dimensions of fatigue: general fatigue, physical fatigue, reduced motivation, reduced activity, and mental fatigue. The instrument includes 20 statements that are evaluated on a 5-point Likert Score scale (1 = I completely agree, 5 = I don’t agree at all).

**Minimental State Examination (MMSE)** (27, 28)

The MMSE is a neuropsychological test that assesses cognitive disorders. It’s a widely used tool that permits an initial evaluation of a patient, but also to follow the evolution of the cognitive disorders through time. It contains tasks of spatial and temporal orientation, word registration and recall, attention and calculation, language skill, repetition and complex commands that are scored 0 to 30 points.

**Frontal Assessment Battery** (29, 30)

The FAB is a short cognitive and behavioural six-subtest battery for the screening of a global executive dysfunction. The global performance on these six subtests (conceptualization, mental flexibility, motor programming, sensitivity to interference, inhibitory control and environmental autonomy) gives a composite score summarizing the severity of the dysexecutive syndrome.

**Trail Making Test (TMT)** (31, 32)

The TMT is a neuropsychological test of visual attention and task switching. It consists of two parts (TMT-A and TMT-B), in which the subject is instructed to connect a set of 25 dots as quickly as possible while still maintaining accuracy. The test can provide information about visual search speed, scanning, speed of processing, mental flexibility, as well as executive functioning.

**Cumulative illness rating scale (CIRS)** (33-35)

The CIRS measures the chronic medical illness (morbidity) burden while taking into consideration the severity of chronic diseases in 14 items representing individual body systems. The general rules for severity rating are: 0 = no problem, 1 = mild problem, 2 = requires first line therapy, 3 = severe problem, 4 = immediate treatment required.

**References**

1. Capitani E, Laiacona M. Composite neuropsychological batteries and demographic correction: standardization based on equivalent scores, with a review of published data. The Italian Group for the Neuropsychological Study of Ageing. J Clin Exp Neuropsychol. 1997;19(6):795-809. doi: 10.1080/01688639708403761.

2. Wall JC, Bell C, Campbell S, Davis J. The Timed Get-up-and-Go test revisited: measurement of the component tasks. J Rehabil Res Dev. 2000;37(1):109-13. doi:

3. Jahantabi-Nejad S, Azad A. Predictive accuracy of performance oriented mobility assessment for falls in older adults: A systematic review. Med J Islam Repub Iran. 2019;33:38. doi: 10.34171/mjiri.33.38.

4. Tinetti ME. Performance-oriented assessment of mobility problems in elderly patients. J Am Geriatr Soc. 1986;34(2):119-26. doi: 10.1111/j.1532-5415.1986.tb05480.x.

5. Guralnik JM, Seeman TE, Tinetti ME, Nevitt MC, Berkman LF. Validation and use of performance measures of functioning in a non-disabled older population: MacArthur studies of successful aging. Aging (Milano). 1994;6(6):410-9. doi:

6. Pavasini R, Guralnik J, Brown JC, et al. Short Physical Performance Battery and all-cause mortality: systematic review and meta-analysis. BMC Medicine. 2016;14(1):215. doi: 10.1186/s12916-016-0763-7.

7. Cruz-Jentoft AJ, Bahat G, Bauer J, et al. Sarcopenia: revised European consensus on definition and diagnosis. Age Ageing. 2018. doi: 10.1093/ageing/afy169.

8. Innes E. Handgrip strength testing: A review of the literature. Australian Occupational Therapy Journal 1999;46:120-40. doi:

9. Marino M, Nicholas JA, Gleim GW, Rosenthal P, Nicholas SJ. The efficacy of manual assessment of muscle strength using a new device. Am J Sports Med. 1982;10(6):360-4. doi: 10.1177/036354658201000608.

10. Benfica PDA, Aguiar LT, Brito SAF, Bernardino LHN, Teixeira-Salmela LF, Faria C. Reference values for muscle strength: a systematic review with a descriptive meta-analysis. Braz J Phys Ther. 2018;22(5):355-69. doi: 10.1016/j.bjpt.2018.02.006.

11. Mudge S, Taylor D, Chang O, Wong R. Test-retest reliability of the StepWatch Activity Monitor outputs in healthy adults. J Phys Act Health. 2010;7(5):671-6. doi:

12. Tudor-Locke C, Bassett DR, Jr. How many steps/day are enough? Preliminary pedometer indices for public health. Sports Med. 2004;34(1):1-8. doi:

13. Tudor-Locke C, Washington TL, Hart TL. Expected values for steps/day in special populations. Prev Med. 2009;49(1):3-11. doi: 10.1016/j.ypmed.2009.04.012.

14. Busse ME, Pearson OR, Van Deursen R, Wiles CM. Quantified measurement of activity provides insight into motor function and recovery in neurological disease. J Neurol Neurosurg Psychiatry. 2004;75(6):884-8. doi: 10.1136/jnnp.2003.020180.

15. Kodraliu G, Mosconi P, Groth N, et al. Subjective health status assessment: evaluation of the Italian version of the SF-12 Health Survey. Results from the MiOS Project. J Epidemiol Biostat. 2001;6(3):305-16. doi:

16. Ware J, Jr., Kosinski M, Keller SD. A 12-Item Short-Form Health Survey: construction of scales and preliminary tests of reliability and validity. Med Care. 1996;34(3):220-33. doi:

17. Ware J, Kosinski MA, Keller SD. SF-12: How to Score the SF-12 Physical and Mental Health Summary Scales. 1998. doi:

18. Mahoney FI, Barthel DW. Functional Evaluation: The Barthel Index. Md State Med J. 1965;14:61-5. doi:

19. Sinoff G, Ore L. The Barthel activities of daily living index: self-reporting versus actual performance in the old-old (> or = 75 years). J Am Geriatr Soc. 1997;45(7):832-6. doi: 10.1111/j.1532-5415.1997.tb01510.x.

20. Nouri FM, Lincoln NB. An extended activities of daily living scale for stroke patients. Clinical Rehabilitation. 1987;1:301-5. doi:

21. Huskisson EC. Measurement of pain. Lancet. 1974;2(7889):1127-31. doi: 10.1016/s0140-6736(74)90884-8.

22. Hirschfeld G, Zernikow B. Variability of "optimal" cut points for mild, moderate, and severe pain: neglected problems when comparing groups. Pain. 2013;154(1):154-9. doi: 10.1016/j.pain.2012.10.008.

23. D'Ath P, Katona P, Mullan E, Evans S, Katona C. Screening, detection and management of depression in elderly primary care attenders. I: The acceptability and performance of the 15 item Geriatric Depression Scale (GDS15) and the development of short versions. Fam Pract. 1994;11(3):260-6. doi: 10.1093/fampra/11.3.260.

24. Galeoto G, Sansoni J, Scuccimarri M, et al. A Psychometric Properties Evaluation of the Italian Version of the Geriatric Depression Scale. Depress Res Treat. 2018;2018:1797536. doi: 10.1155/2018/1797536.

25. Hagelin CL, Wengstrom Y, Runesdotter S, Furst CJ. The psychometric properties of the Swedish Multidimensional Fatigue Inventory MFI-20 in four different populations. Acta Oncol. 2007;46(1):97-104. doi:

26. Smets EM, Garssen B, Bonke B, De Haes JC. The Multidimensional Fatigue Inventory (MFI) psychometric qualities of an instrument to assess fatigue. J Psychosom Res. 1995;39(3):315-25. doi:

27. Folstein MF, Folstein SE, McHugh PR. "Mini-mental state". A practical method for grading the cognitive state of patients for the clinician. J Psychiatr Res. 1975;12(3):189-98. doi:

28. Measso G, Cavarzeran F, Zappala G, et al. The mini‐mental state examination: Normative study of an Italian random sample. Developmental Neuropsychology. 1993;9(2):77-85. doi:

29. Appollonio I, Leone M, Isella V, et al. The Frontal Assessment Battery (FAB): normative values in an Italian population sample. Neurol Sci. 2005;26(2):108-16. doi: 10.1007/s10072-005-0443-4.

30. Dubois B, Slachevsky A, Litvan I, Pillon B. The FAB: a Frontal Assessment Battery at bedside. Neurology. 2000;55(11):1621-6. doi: 10.1212/wnl.55.11.1621.

31. Bowie CR, Harvey PD. Administration and interpretation of the Trail Making Test. Nat Protoc. 2006;1(5):2277-81. doi: 10.1038/nprot.2006.390.

32. Tombaugh TN. Trail Making Test A and B: normative data stratified by age and education. Arch Clin Neuropsychol. 2004;19(2):203-14. doi: 10.1016/S0887-6177(03)00039-8.

33. Hudon C, Fortin M, Soubhi H. Abbreviated guidelines for scoring the Cumulative Illness Rating Scale (CIRS) in family practice. J Clin Epidemiol. 2007;60(2):212. doi: 10.1016/j.jclinepi.2005.12.021.

34. Miller MD, Paradis CF, Houck PR, et al. Rating chronic medical illness burden in geropsychiatric practice and research: application of the Cumulative Illness Rating Scale. Psychiatry Res. 1992;41(3):237-48. doi: 10.1016/0165-1781(92)90005-n.

35. Kirkhus L, Jordhoy M, Saltyte Benth J, et al. Comparing comorbidity scales: Attending physician score versus the Cumulative Illness Rating Scale for Geriatrics. J Geriatr Oncol. 2016;7(2):90-8. doi: 10.1016/j.jgo.2015.12.003.

**Additional file 2**: Box Plots of gait parameters

| 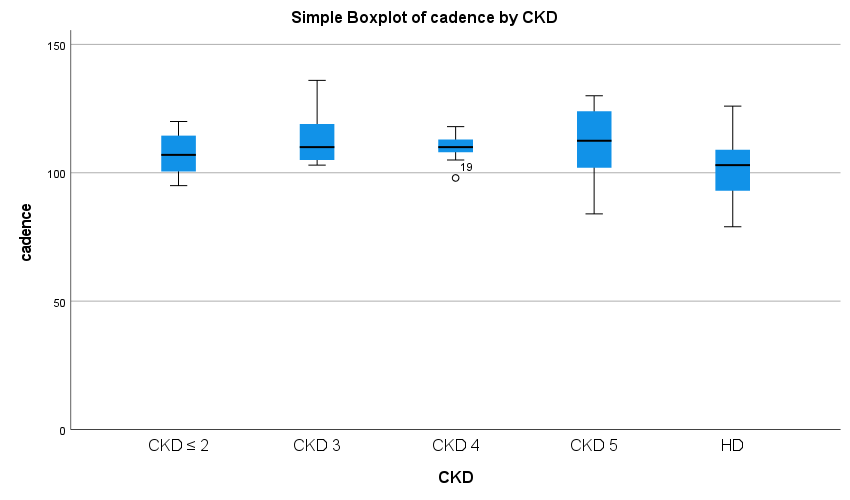 | 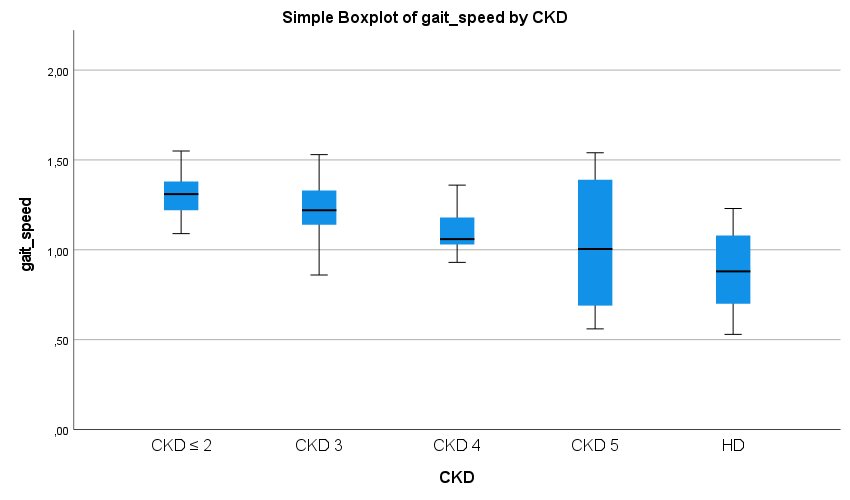 |
| --- | --- |
| 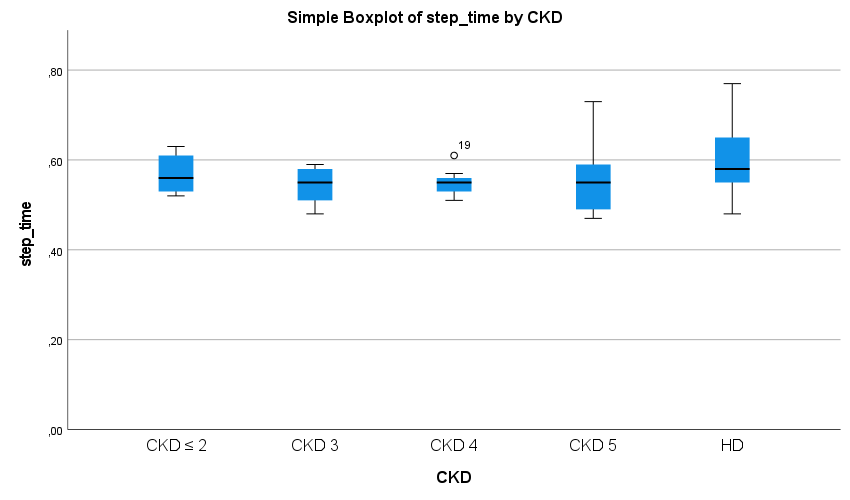 | 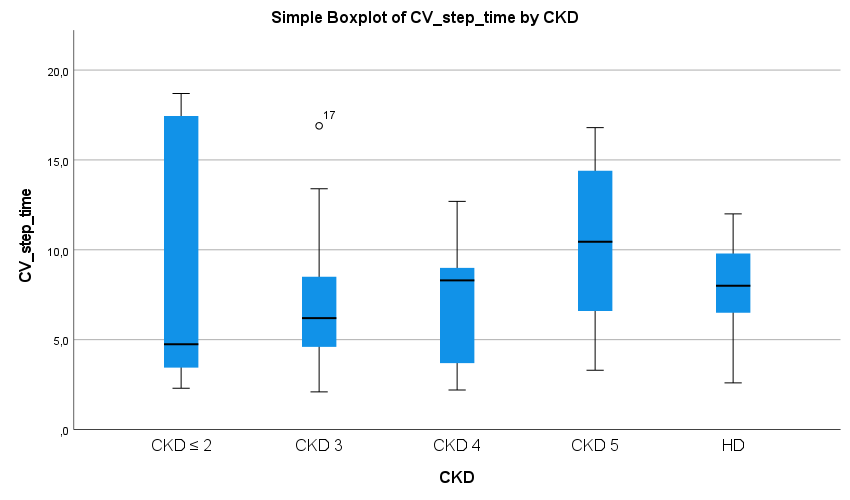 |
| 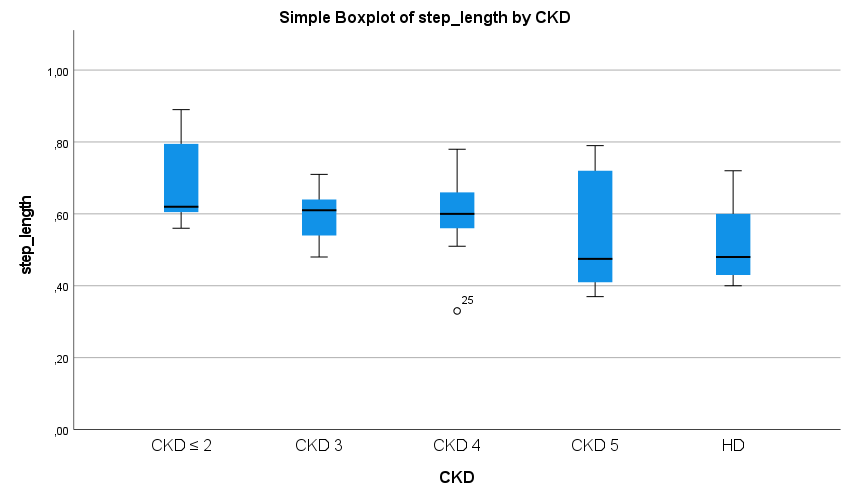 | 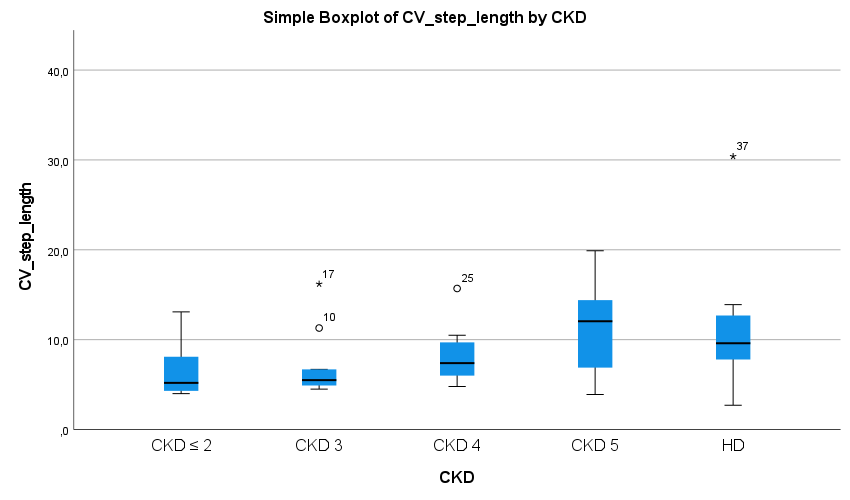 |
| 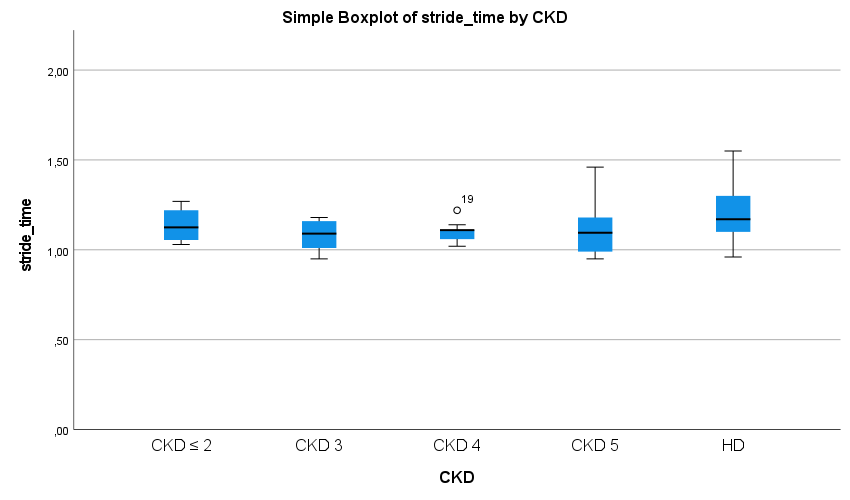 | 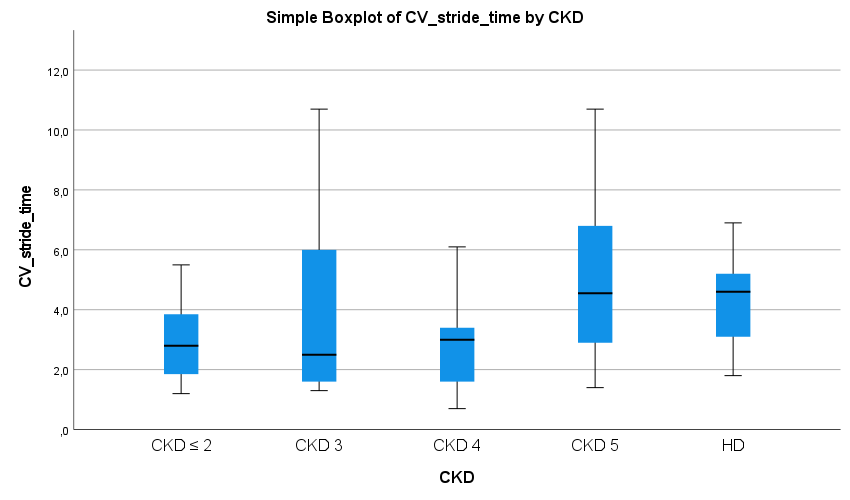 |
| 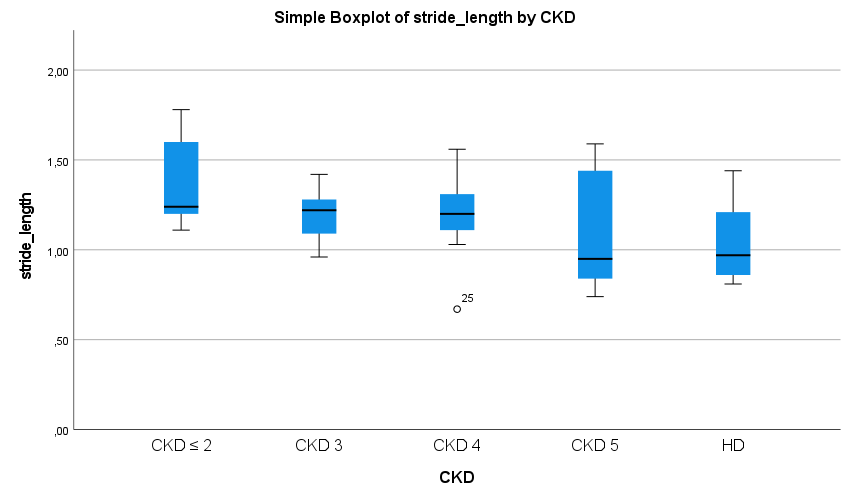 | 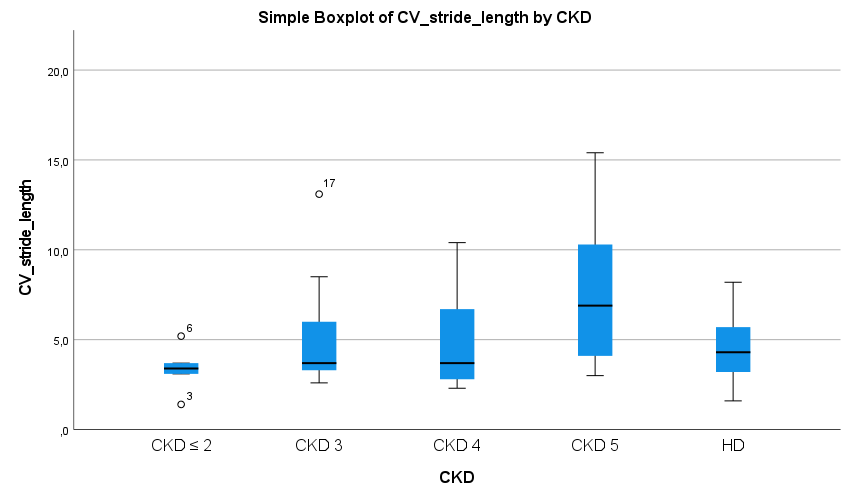 |
| 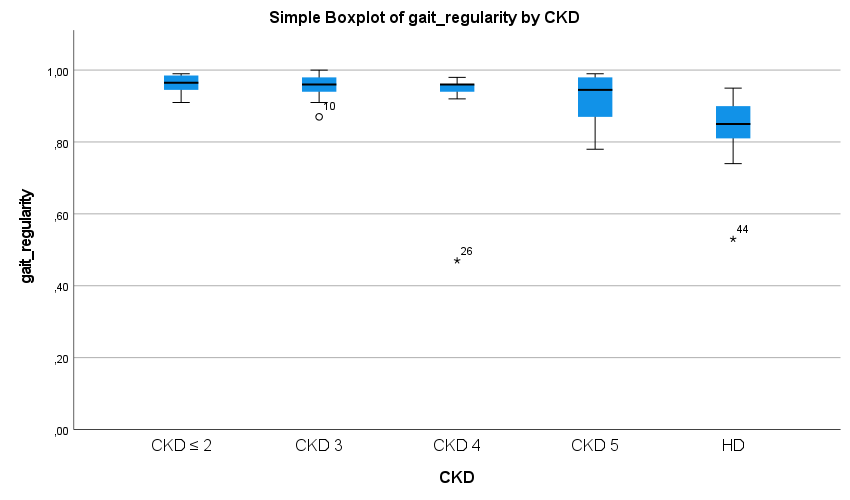 |  |

**Additional file 3:** STROBE Statement – Checklist of items that should be included in reports of cross-sectional studies

|  | Item No. | Recommendation | Page  No. | Relevant text from manuscript |
| --- | --- | --- | --- | --- |
| **Title and abstract** | 1 | (*a*) Indicate the study’s design with a commonly used term in the title or the abstract | 1 |  |
|  |  | (*b*) Provide in the abstract an informative and balanced summary of what was done and what was found | 3 |  |
| Introduction | | | |  |
| Background/rationale | 2 | Explain the scientific background and rationale for the investigation being reported | 3-4 |  |
| Objectives | 3 | State specific objectives, including any prespecified hypotheses | 4 |  |
| Methods | | | |  |
| Study design | 4 | Present key elements of study design early in the paper | 4 |  |
| Setting | 5 | Describe the setting, locations, and relevant dates, including periods of recruitment, exposure, follow-up, and data collection | 4-5 |  |
| Participants | 6 | (*a*) *Cohort study*—Give the eligibility criteria, and the sources and methods of selection of participants. Describe methods of follow-up  *Case-control study*—Give the eligibility criteria, and the sources and methods of case ascertainment and control selection. Give the rationale for the choice of cases and controls  ***Cross-sectional study*—Give the eligibility criteria, and the sources and methods of selection of participants** | 5 |  |
|  |  | (*b*) *Cohort study*—For matched studies, give matching criteria and number of exposed and unexposed  *Case-control study*—For matched studies, give matching criteria and the number of controls per case | NA |  |
| Variables | 7 | Clearly define all outcomes, exposures, predictors, potential confounders, and effect modifiers. Give diagnostic criteria, if applicable | 5 |  |
| Data sources/ measurement | 8* | For each variable of interest, give sources of data and details of methods of assessment (measurement). Describe comparability of assessment methods if there is more than one group | 5 / Table 1 |  |
| Bias | 9 | Describe any efforts to address potential sources of bias | NA |  |
| Study size | 10 | Explain how the study size was arrived at | 6 |  |

Continued on next page

| Quantitative variables | 11 | Explain how quantitative variables were handled in the analyses. If applicable, describe which groupings were chosen and why | 6 |  |
| --- | --- | --- | --- | --- |
| Statistical methods | 12 | (*a*) Describe all statistical methods, including those used to control for confounding | 6 |  |
|  |  | (*b*) Describe any methods used to examine subgroups and interactions | 6 |  |
|  |  | (*c*) Explain how missing data were addressed | 6 |  |
|  |  | (*d*) *Cohort study*—If applicable, explain how loss to follow-up was addressed  *Case-control study*—If applicable, explain how matching of cases and controls was addressed  ***Cross-sectional study*—If applicable, describe analytical methods taking account of sampling strategy** | NA |  |
|  |  | (*e*) Describe any sensitivity analyses | 6 |  |
| Results | | | | |
| Participants | 13* | (a) Report numbers of individuals at each stage of study—eg numbers potentially eligible, examined for eligibility, confirmed eligible, included in the study, completing follow-up, and analysed | 6 |  |
|  |  | (b) Give reasons for non-participation at each stage | NA |  |
|  |  | (c) Consider use of a flow diagram | NA |  |
| Descriptive data | 14* | (a) Give characteristics of study participants (e.g. demographic, clinical, social) and information on exposures and potential confounders | Table 1 |  |
|  |  | (b) Indicate number of participants with missing data for each variable of interest | 6 / Table 2 |  |
|  |  | (c) *Cohort study*—Summarise follow-up time (e.g., average and total amount) | NA |  |
| Outcome data | 15* | *Cohort study*—Report numbers of outcome events or summary measures over time | NA |  |
|  |  | *Case-control study—*Report numbers in each exposure category, or summary measures of exposure | NA |  |
|  |  | ***Cross-sectional study—*Report numbers of outcome events or summary measures** | 6-7 |  |
| Main results | 16 | (*a*) Give unadjusted estimates and, if applicable, confounder-adjusted estimates and their precision (e.g., 95% confidence interval). Make clear which confounders were adjusted for and why they were included | Table 3 |  |
|  |  | (*b*) Report category boundaries when continuous variables were categorized | NA |  |
|  |  | (*c*) If relevant, consider translating estimates of relative risk into absolute risk for a meaningful time period | NA |  |

Continued on next page

| Other analyses | 17 | Report other analyses done—eg analyses of subgroups and interactions, and sensitivity analyses | Table 3 |  |
| --- | --- | --- | --- | --- |
| Discussion | | | | |
| Key results | 18 | Summarise key results with reference to study objectives | 7 |  |
| Limitations | 19 | Discuss limitations of the study, taking into account sources of potential bias or imprecision. Discuss both direction and magnitude of any potential bias | 9 |  |
| Interpretation | 20 | Give a cautious overall interpretation of results considering objectives, limitations, multiplicity of analyses, results from similar studies, and other relevant evidence | 8-9 |  |
| Generalisability | 21 | Discuss the generalisability (external validity) of the study results | 8-9 |  |
| Other information | |  | | |
| Funding | 22 | Give the source of funding and the role of the funders for the present study and, if applicable, for the original study on which the present article is based | 11 |  |

*Give information separately for cases and controls in case-control studies and, if applicable, for exposed and unexposed groups in cohort and cross-sectional studies.

**Note:** An Explanation and Elaboration article discusses each checklist item and gives methodological background and published examples of transparent reporting. The STROBE checklist is best used in conjunction with this article (freely available on the Web sites of PLoS Medicine at http://www.plosmedicine.org/, Annals of Internal Medicine at http://www.annals.org/, and Epidemiology at http://www.epidem.com/). Information on the STROBE Initiative is available at www.strobe-statement.org.
